# Comparison of Recommendations Made by Committee Members with and without Financial Conflict of Interest on Japanese Guideline of Treatment of Hyperuricemia and Gout, Third Edition

**DOI:** 10.31662/jmaj.2023-0067

**Published:** 2023-09-25

**Authors:** Ichiro Hisatome, Toshihiro Hamada, Einosuke Mizuta, Akira Ohtahara, Masanari Kuwabara, Kazuhide Ogino, Haruaki Ninomiya, Yasuto Sato, Takeo Nakayama, Hisashi Yamanaka

**Affiliations:** 1 Department of Cardiology, National Hospital Organization, Yonago Medical Center, Yonago, Japan; 2 Department of Community-based Family Medicine, Tottori University Faculty of Medicine, Yonago, Japan; 3 Department of Cardiology, Sanin Rosai Hospital, Yonago, Japan; 4 Department of Cardiology, Toranomon Hospital, Tokyo, Japan; 5 Department of Cardiology, Japanese Red Cross Tottori Hospital, Tottori, Japan; 6 Department of Biological Regulation, Tottori University Faculty of Medicine, Yonago, Japan; 7 Graduate School of Public Health, Shizuoka Graduate University of Public Health, Shizuoka, Japan; 8 Department of Health Informatics, Graduate School of Medicine & School of Public Health, Kyoto University, Kyoto, Japan; 9 Department of Rheumatology, Sanno Medical Center, Tokyo, Japan

**Keywords:** conflict of interest, clinical guideline, hyperuricemia, gout

## Abstract

Clinical practice guidelines (CPGs) consist of clinical questions (CQs) and corresponding recommendations. Considering the estimation of body of evidence, patients’ opinions, and medical economics, recommendations can vary depending on the votes of the committee members of CPGs. Taking this into consideration, concerns have already been raised on how financial conflict of interest (COI) potentially influences recommendations. In this study, we developed the third edition of guideline for the management of hyperuricemia and gout. This CPG was composed of seven CQs and recommendations. The direction and strength of the recommendations were determined by votes. There are three CQs. Individual questions asked whether uric acid-lowering-agents (ULAs) could be applied to hyperuricemic patients with chronic kidney disease (CKD) (CQ A), hypertension (CQ B), or heart failure (CQ C) to prevent organ damage. We examined whether the absence (18 members) or presence (8 members) of COIs of committee members could influence the votes. In total, 26 committee members with and without COI have equally determined the direction and strength of recommendations. In CQ A, members without financial COIs and those with financial COI selected conditional recommendation for the use of ULAs in patients with CKD (without COI, 17/18; with COI, 7/8). In CQ B, members without financial COIs and those with financial COI selected conditional recommendation against the use of ULAs in hypertensive patients (without COI, 14/18; with COI, 5/8). In CQ C, members without financial COIs and those with financial COIs have selected conditional recommendation against the use of ULAs in patients suffering from heart failure (without COI, 15/18; with COI, 4/8).

We found that members with financial COIs have determined their recommendations in the same direction and strength as those without financial COIs.

Clinical practice guidelines (CPGs) for the management of diseases are recently being developed worldwide, to offer standard diagnosis and treatment. CPGs are identified to be important tools for appropriate patient care. They include recommendations intended to optimize patient care and assessment of benefits and harms of alternative care options. Basically, CPGs are composed of clinical questions (CQs) and their corresponding recommendations. In brief, based on systematic reviews (SR) of reports related to the outcome of each CQ, the body of evidence is evaluated by factors of domains including bias risk, inconsistency, imprecision, indirectness, and publication bias. Considering body of evidence, patients’ preferences, and medical economics, recommendations in response to CQs are determined by means of votes by the committee members of CPG.

Conflict of interest (COI) potentially influences the development of CPGs. A COI is defined as a set of conditions in which professional judgment is of primary interest (such as health and well-being of a patient or validity of research) and is influenced by a secondary interest ^(1)^ involving financial and nonfinancial interest. Nonfinancial interest is usually composed of research activity, individual expertise, promotion, and human relationship. Therefore, in order to determine the recommendations in response to CQs in CPGs, disclosure of financial and nonfinancial COI, especially ties of the committee to pharmaceutical companies, is of great important. Recommendations to CQs in CPGs are primarily based on the evaluation of the body of evidence by means of meta-analysis. COI may influence the judgment of balance between outcomes of benefits and harms induced by intervention, resulting in overestimation of benefits and/or underestimation of harms. Thus, COI of the members should be appropriately managed.

It has been reported that COI could influence recommendation in CPGs ^(2)^. The effect of COI was confined to case studies showing that authors with specific financial ties appeared to benefit the related CPG recommendations. It has also been reported that meta-analyses on antihypertensive drugs with financial ties to one drug company were associated with favorable conclusion ^(3)^. However, it remains to be elucidated whether recommendations to CQs could be influenced by the absence or presence of financial COI of members.

Evidences have been accumulated showing that hyperuricemia could be an independent risk factor not only for gout and urolithiasis but also for renal failures and cardiovascular events. In CPG for the management of hyperuricemia and gout second edition published in 2010, hyperuricemia was defined as a risk factor and marker for organ damage and life-related disease. Since the CQs in the second edition were expressed by PICO format without harmful outcome, the evaluation of balance between advantage and disadvantage outcomes was absence. The third edition of the guideline for the management of hyperuricemia and gout was published in 2018, which was based on a systematic review conducted in 2017 ^(4)^. Seven CQs accompanied by advantage and disadvantage outcomes have been selected, and recommendations were determined. In the third edition, the CQs are expressed via PICO format with both beneficial and harmful outcomes to secure the balance between benefit and harm on the treatment of hyperuricemia and gout. In addition, the value set and preference of patients as well as the costs and resources were included. The third edition primarily focused on the possibility whether hyperuricemia could cause organ damage. In this study, we have three CQs asking whether uric acid-lowering agents (ULAs) could be utilized for hyperuricemic patients with either chronic kidney disease (CKD), hypertension, or heart failure. Use of ULAs has been conditionally recommended for hyperuricemic patients with CKD in order to preserve renal function (CQ A). Meanwhile, use of ULAs was conditionally recommended for hyperuricemic patients with hypertension in order to improve the prognosis of cardiovascular events (CQ B). Lastly, use of ULAs was also conditionally recommended for hyperuricemic patients with heart failure in order to improve the prognosis of the cardiovascular events (CQ C) (Table 1).

**Table 1.** 

CQ (A): Can urate-lowering agents be recommended in patients with hyperuricemia and kidney injury over non-medication treatment?		
Recommendation	Direction and strength of the recommendation	Certainty in evidence
The use of urate-lowering agents to retard the decline in kidney function is conditionally recommended in patients with hyperuricemia and kidney injury.	It is conditionally recommended for.	B (moderate)
CQ (B): Can urate-lowering agents be recommended for hypertensive patients with hyperuricemia over non-medication treatment?		
Recommendation	Direction and strength of recommendation	Certainty in evidence
The use of urate-lowering agents to improve life prognosis and reduce the risk of cardiovascular disease cannot be actively recommended for hypertensive patients with hyperuricemia	It is conditionally recommended against.	D (very weak)
CQ (C): Can urate-lowering agents be recommended for patients with heart failure and hyperuricemia over non-medication treatment?		
Recommendation	Direction and strength of recommendation	Certainty in evidence
The use of urate-lowering agents to improve life prognosis and reduce the risk of cardiovascular disease cannot be actively recommended for patients with heart failure and hyperuricemia.	It is conditionally recommended against.	C (weak)

Recommendations commonly have two elements, direction and strength. The direction of the recommendation refers to whether the recommendation will be implemented or not. Meanwhile, the strength of the recommendation refers to whether the recommendation is strong or weak, depending on the certainty of evidence which corresponds to A (strong), B (moderate), C (weak/low), or D (very weak/low) according to the Grading of Recommendations, Assessment, Development, and Evaluations (GRADE) approach ^(5)^. Evaluating the certainty of the body of evidence is often conducted based on factors of domains. Recommendations were determined based on the votes provided by 26 committee members (8 cardiologists, 2 nephrologists, 1 pharmacist, 5 diabetologists, 2 public health experts, 5 rheumatologists, 2 hematologists, and 1 urologist). According to the rules of participation, those who received an amount of money exceeding JPY 500,000 but less than JPY 5 million from industrial companies could participate in the deliberation, but could not participate in the voting. As the three CQs of this study have asked the safety and effectivity of the usage of ULAs to suppress renal damage or cardiovascular event, the recommendations in response to these CQs could be affected by committee members’ financial ties with pharmaceutical companies producing ULAs. In total, 8 out of 26 guideline committee members had financial COIs regarding these three CQs and could not participate in the voting. Thus, recommendations regarding these CQs were determined by the remaining 18 members without COIs. Besides the 18 members without COI, 8 members with COI were asked to vote on the three CQs experimentally, in order to compare the direction and strength of recommendations toward each CQ between members without COIs and those with COIs. Those with COIs knew that their votes were not reflected on the determination but had been analyzed afterward.

Table 2 shows the result of votes by 18 members without COIs and 8 members with COIs. In CQ A, conditional recommendation “against the use of drugs as a treatment” got 1 vote (5.6%), and that “for the use of drugs as a treatment” got 17 votes (94.4%) obtained from votes by 18 members without COIs. In the votes by 8 members with COI, the conditional recommendation “against the use of drugs as a treatment” got 1 vote (12.5%), and that “for the use of drugs as a treatment” got 7 votes (87.5%), indicating the same results for members without COIs. In CQ B, the recommendation “against the use of drugs as a treatment” got 2 votes (11.1%), the conditional recommendation “against the use of drugs as a treatment” got 14 votes (77.8%), and the conditional recommendation “for the use of drugs as a treatment” got 2 votes (11.1%), as obtained from the votes conducted by the 18 members without COIs. As per the votes by 8 members with COI, the conditional recommendation “against the use of drugs as a treatment” got 5 votes (62.5%), and that “against the use of drugs as a treatment” got 3 votes (37.5%), indicating the increase in recommendation “against the use of drugs as a treatment.” In CQ C, the recommendation “against the use of drug as a treatment” got 2 votes (11.1%), the conditional recommendation “against the use of drugs as a treatment” got 15 votes (83.3%), and the conditional recommendation “for the use of drugs as a treatment” got 1 vote (5.6%), as obtained from the votes by 18 members without COIs. Meanwhile, as per the votes by 8 members with COI, the recommendation “against the use of drugs as a treatment” got 4 votes (50%), and the conditional recommendation “against the use of drugs as a treatment” got 4 votes (50%), indicating the increase in the recommendation “against the use of drugs as a treatment.”

**Table 2.** 

			Direction and strength of recommendation			
CQ A		Total	Recommended against	Conditionally recommended against	Conditionally recommended for	Recommended for
	COI (−)	18	0	1 (5.6%)	17(94.4%)	0
	COI (+)	8	0	1(12.5%)	7(87.5%)	0
			Direction and strength of recommendation			
CQ B		Total	Recommended against	Conditionally recommended against	Conditionally recommended for	Recommended for
	COI (−)	18	2 (11.1%)	14 (77.8%)	2 (11.1%)	0
	COI (+)	8	3 (37.5%)	5 (62.5%)	0	0
			Direction and strength of recommendation			
CQ C		Total	Recommended against	Conditionally recommended against	Conditionally recommended for	Recommended for
	COI (−)	18	2 (11.1%)	15 (83.3%)	1 (5.6%)	0
	COI (+)	8	4 (50%)	4 (50%)	0	0

It has been suggested using SR that most guideline developers disclose financial COIs with pharmacological companies, with estimates between 56% and 87%, indicating that the guideline developers had financial ties to pharmaceutical industries. Payments coming from any pharmaceutical industry have been associated with changes in recommendations in ways that are favorable to the industry. Evidence provokes the call for chairs and majority of developers to be free of financial ties and recommendation for exclusion of any conflicted developers. However, there is few reports that clarified whether recommendations in CQs could be influenced by the absence or presence of financial COI of members or not. In this present report, we examined the effects of financial COI on the direction and strength of recommendations by votes of the members and found that the members with COIs showed the same direction and similar or even stronger strength of their recommendation against the use of the drug as members without COIs. This is the first report showing that in the votes for recommendation, COIs did not alter the direction and strength of recommendation toward CQs favorably for related companies. This may be because the members with COIs make strict judgment on recommendations for each CQ because of their expertise. However, it is speculated that members with financial COI might make rather strict judgment in order to avoid suspicious involvement in financial interest, since they knew that their votes had not been reflected on the determination but had been analyzed afterward.

There is a limitation including that COIs in this committee members correspond to financial COIs alone; thus, we could not tell whether nonfinancial COIs may influence the result or not. Further research is required to clarify this point. We could not determine if our present finding might be applicable to other CPGs; however, we can conclude that the presence of financial COIs alone may not always influence the determination of the recommendation in CPGs.

## Article Information

### Conflicts of Interest

IH received research grants from Mochida Pharmaceutical, Meiji, and Fujiyakuhin and lecture fees from Mochida Pharmaceutical, Fujiyakuhin, Sanawa kagaku, Teijin Pharma, Taisho Pharmaceutical, Sato Seiyaku, and Boehringer Ingelheim.

TN received research grants from I&H Co., Ltd., Cocokarafine Group Co., Ltd., and Konica Minolta Inc.; consulting fees from Otsuka Pharmaceutical; honoraria from Pfizer Japan INC., MSD K.K., Chugai Pharmaceutical Co., Takeda Pharmaceutical Co., Janssen Pharmaceutical K.K., Boehringer Ingelheim International GmbH, Eli Lilly Japan K.K., Maruho Co., Ltd., Mitsubishi Tanabe Pharma Corporation, Novartis Pharma K.K., Allergan Japan K.K., Novo Nordisk Pharma Ltd., TOA EIYO Ltd., Dentsu Group Inc., ONO PHARMACEUTICAL CO., LTD., GSK plc, Alexion Pharmaceuticals, Inc., and Canon Medical Systems Co.; stock options from Bon Bon Inc.; and donations from CancerSCAN and YUYAMA Co.

HY received honoraria from Teijin Pharma, Fuji Yakuhin, Mochida Pharmaceutical, and Sato Seiyaku.

### Author Contributions

・Concept: Ichiro Hisatome and Hisashi Yamanaka

・Data collection: Toshihiro Hamada, Einosuke Mizuta, Akira Ohtahara, Masanari Kuwabara, and Kazuhide Ogino

・Discussion: Haruaki Ninomiya, Yasuto Sato, and Takeo Nakayama
